# Endovascular treatment of pediatric vertebral artery dissection: Case report and literature review

**DOI:** 10.1016/j.ijscr.2025.111935

**Published:** 2025-09-15

**Authors:** Yi Zhang, Yujun Liao, Bin Xu, Yanlong Tian, Hao Li

**Affiliations:** a Department of Neurosurgery, Children's Hospital of Fudan University, Shanghai, 201102, China; b Department of Neurosurgery, Fudan University Huashan Hospital, Shanghai, 200040, China

**Keywords:** Vertebral artery dissection, Endovascular treatment, Pediatric

## Abstract

**Introduction and importance:**

Vertebral artery dissection (VAD) is considered a rare disease in the pediatric population, with challenging to diagnose and treatment. The optimal management remains unclear. Outcome of endovascular treatment, is still rarely reported. Along with a literature review, we aimed to provide information of clinical manifestation, treatment and prognosis in endovascular treatment of pediatric VAD.

**Presentation of case:**

Two endovascular treated cases of spontaneous pediatric VAD were retrospectively reviewed. Clinical symptoms were only headache. Diagnosis was confirmed by DSA. Endovascular coils occlusions of parental vertebral artery were performed in two cases. Both individuals experienced a full recovery, and there were no further occurrences of symptoms or new infarction.

**Clinical discussion:**

VAD is a rare but significant cause of stroke in children. Its incidence in the pediatric population is low, with males being more frequently affected than females. Treatment options for pediatric VAD include conservative medical management, surgical intervention, and endovascular therapy. Endovascular treatment, though less commonly reported, has shown promise in achieving good clinical outcomes with minimal complications. We report two pediatric cases of VAD treated with endovascular therapy. Both patients presented with headache as the primary symptom and achieved full recovery without neurological deficits. Endovascular treatment appears to be a safe and effective option for pediatric VAD, especially in cases where conservative management is insufficient or surgical intervention is not feasible.

**Conclusions:**

Pediatric VAD is rare and endovascular treatment is feasible for the treatment. More research will still be needed to demonstrate the superiority in clinical outcomes with endovascular therapy.

## Highlights

• We report two pediatric VADs and performed a literature review. It is conducive to the diagnosis and management.
• We compared the outcomes of surgical and endovascular treatment.
• Endovascular treatment of VAD in pediatric population seems to be safe and effective

## 1 Introduction

Vertebral artery dissecting (VAD) is a rare disease, predominantly associated with posterior circulation arterial ischemic stroke (PCAIS) or transient ischemic attack (TIA) in pediatric cases [1]. The limited number of published pediatric VAD cases are frequently reported as isolated incidents or combined with carotid artery dissection cases, which limits the identification of distinct clinical characteristics, therapeutic approaches, and outcomes for this particular group of patients.

Trauma is frequently implicated as a precursor to vertebral artery dissection (VAD) in children, although not every child with VAD has a documented history of trauma [2]. Magnetic resonance angiography (MRA) is typically the preferred method for assessing cerebrovascular circulation in pediatric arterial ischemic stroke (AIS), despite its acknowledged limitations in sensitivity and specificity in cases of PCAIS [3]. While computed tomographic angiography (CTA) has demonstrated high sensitivity in diagnosing vertebral artery dissection (VAD) in adults [4], its efficacy in diagnosing acute ischemic stroke (AIS) in children remains uncertain. In cases involving trauma, CTA may yield false-negative results, particularly when the vertebral artery is obscured at the skull base. As a result, digital subtraction angiography (DSA) continues to be regarded as the gold standard for diagnosing VAD. [1]. At present, anticoagulant therapy is the suggested treatment for extracranial VAD in pediatric patients, however the optimal management of VAD is not established [2]. The assessment of the risks versus benefits of endovascular treatment options remains equivocal [5]. Endovascular interventions for VAD in children have had positive outcomes in a small number of documented cases [[6], [7], [8]]. While the data on complications and clinical results indicate that such treatments are likely both safe and effective, these assessments still require confirmation from a larger cohorts and long-term follow-ups [9].

In this review, we report 2 pediatric cases of VAD with the goal of delineating the clinical manifestations, diagnostic imaging, and discussing the prognosis of endovascular treatment in these patients. This case report has been reported in line with the SCARE checklist [10].

## 2 Case reports

### 2.1 Patient 1

**Fig. 1:**
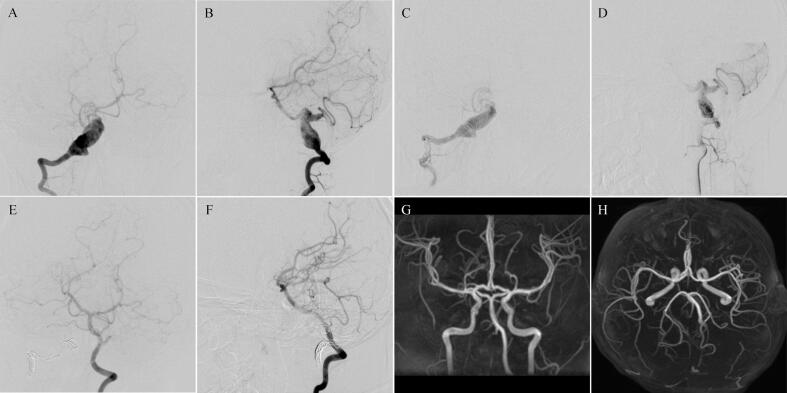
DSA and MRA of Patient 1. Preoperative anteroposterior and lateral position angiograms of the RVA (A, B), showing a giant dissecting aneurysm involved right posterior inferior cerebellar artery. Immediately post-operative angiograms of the RVA showing decreased parent artery flow (C, D). 1-year post-treatment DSA showing complete occlusion of the aneurysm and a dominant LVA (E, F). 1-year post-treatment MRA showing complete occlusion of the aneurysm (G, H). RVA, right vertebral artery; DSA, digital subtraction angiography; LVA, left vertebral artery. MRA, magnetic resonance angiography.

A 10-year-old male had a three-month history of headache symptoms prior to hospitalization, which initially presented as a continuous bilateral frontal headache and increased in last 1 week. Upon admission, Physical examinations were normal and with no signs of dysphagia and dysarthria. The cranial nerve examination is unremarkable.MR and MRA study found a nodule at right anterior of the medulla, raising concern for right vertebral artery aneurysm with possible thrombosis. A diagnostic digital subtraction angiography (DSA) was performed for evaluation of the bilateral vertebral arteries, internal and external carotid. DSA revealed a non-dominant right vertebral artery alongside a dominant left vertebral artery, and demonstrated the focal area of right V3 and V4 segments dilation and irregularity (Fig. 1A, B).

The endovascular treatments were performed under general anesthesia. After canalizing the femoral artery with a 6-F arterial sheath, we placed a 6-F guiding catheter (Fargomax, Balt) in the distal V2 segment of the right vertebral artery (RVA). Using the road map, we advanced a micro catheter (Echelon-10, eV3) to the V3 segment of RVA. The artery was subsequently occluded endovascularly by using multiple coils (Axium, eV3) at the level of V3. The procedure was proceeded smoothly without any complications (Fig. 1C-F). The patient tolerated the procedure well, with no change in neurological examination during or after the coiling. The patient returned for a follow-up at 1-year post-embolization. There were no reports of new symptoms or evidence of infarction. The post-embolization DSA shown complete occlusion of the aneurysm and a dominant left vertebral artery (LVA). MRA confirmed the occlusion of the right vertebral artery and the maintained flow within the collateral circulation of the LVA (Fig. 1G, H).

### 2.2 Patient 2

**Fig. 2:**
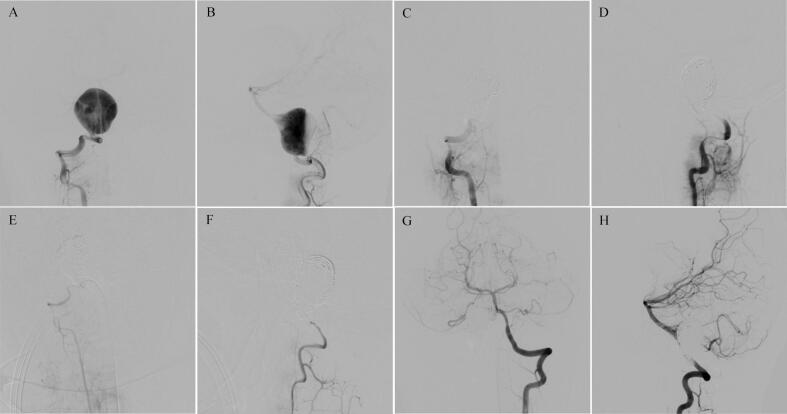
DSA of Patient 2. Preoperative anteroposterior and lateral position angiograms of the RVA (A, B), showing a giant dissecting aneurysm. Immediately post-operative angiograms of the RVA showing complete occlusion of the aneurysm (C, D). 1-year post-treatment DSA of the RVA showing complete occlusion of the aneurysm and the occluded RVA is thinner, (E, F). Angiography also showed a dominant LVA and right anterior inferior cerebellar artery has good blood flow from the angiography of LVA (G, H). RVA, right vertebral artery; DSA, digital subtraction angiography; LVA, left vertebral artery.

A 10-year-old male presented with occipital headache, accompanied by nausea, vomiting, and gait disturbances attributed to vertigo. Upon initial assessment, neurological function was intact with no signs of ataxia or facial nerve palsy. A CT image of the brain was obtained, showing anterior high-density (44–58 HU) mass of brainstem. Contrast MR and MRA found vertebral artery dissecting with aneurysm formation. A diagnostic DSA was performed and demonstrated the focal area of right V3 segments dissecting with aneurysm formation (Fig. 2A, B). DSA also showed a non-dominant right vertebral artery alongside a dominant left vertebral artery.

The endovascular treatments were performed under general anesthesia. After canalizing the femoral artery with a 6-F arterial sheath, we placed a 6-F guiding catheter (Fargomax, Balt) in the distal V2 segment of the right vertebral artery (RVA). Using the road map, we advanced two micro catheters (Echelon-10, eV3) to the V3-V4 segment of RVA. The artery was subsequently occluded endovascularly by using multiple coils (Axium, eV3) at the level of V4-V5. The patient had an endovascular coils occlusion procedure to occlude both the aneurysm and its associated blood vessel (Fig. 2C, D). Following the procedure, the patient experienced no additional strokes. The patient remained symptom-free and exhibited no neurological impairments at 1-year follow-up. The post-embolization DSA showed that the abnormal segment of the vertebral artery and the dissecting aneurysms were fully occluded by the coils (Fig. 2E-H).

## 3 Discussion

The overall incidence of stroke in children is around 2.5–2.7 per 100,000 [11]. Specifically, cerebral artery dissection is an infrequent, challenging-to-diagnose, and potentially underappreciated etiology of ischemic stroke, accounting for 7.5 to 20 % of such cases [12]. Available date concerning vertebral artery dissection and their treatment and clinical outcome are more limited [[13], [14], [15]]. In this case series, we reported two VAD cases in two 10-year-old boys and retrieved 6 cases of endovascular treated pediatric VAD, including information of clinical symptom, treatment and outcome, from the existing literature sources.

Izhar et al. [2] reviewed 68 pediatric cases of VAD. Males were 6.6 times more likely to be affected than females. The predominant clinical manifestations in this pediatric review included eye movement disorders (72 %), weakness or paralysis in one or more limbs (54 %), headaches (38 %), ataxia (53 %), vomiting (37 %), and episodes of unconsciousness (34 %). Hina et al. [16] documented 20 cases of extracranial vertebral artery dissection in children through a retrospective single-center study spanning 14 years. There was a notable male preponderance (sex ratio of 9/1). Initial symptoms included headaches (45 %), neck pain (15 %), nausea (30 %), and vertigo (30 %). Clinical signs that prompted hospital admission were hemiparesis (60 %), visual disturbances with oculomotor dysfunction (20 %) or visual field defects (20 %), and cerebellar syndrome (35 %). Our 2 patients and 6 cases from literatures demonstrate a remarkable sex predilection of cases. All patients were male. Our case presented with milder clinical phenotype, only with symptoms of a headache and no neurological deficit. Cases from literatures showed similar clinical features to previous reports.

It remains unclear which imaging technique is the most sensitive for detecting VAD in children with suspected cases. In the report by Hina et al. [16], the initial diagnostic imaging for vertebral artery dissection was conducted using DSA for 11 patients (55 %), MRA for 7 patients (35 %), and CT angiography for 2 patients (10 %). In Izhar Hasan's report [2], Angiography was conducted on 63 out of the 68 pediatric patients with VAD. A systematic review has indicated that MRI combined with MRA is less sensitive for diagnosing VAD compared to catheter angiography or CTA. [17]. Mackay et al. [18] also documented instances where VAD was identified on subsequent catheter angiography, indicating a recurrent course in some cases. Both our patients and the 6 reported cases in recent literatures performed catheter angiography for VAD diagnosis. Considering the safety of catheter angiography when performed by skilled practitioners and the critical nature of establishing a diagnosis for prognostic and therapeutic purposes, we believe there is a compelling argument for utilizing catheter angiography in pediatric cases of VAD.

The V2-V3 segment was the most frequently affected part of the artery [16]. In our patients, the V3 segment was the affected segment in both cases. VAD was predominantly located at the transition between the atlas and axis, which is the uppermost portion of the V2 segment, likely because of the significant mobility of the artery at this point [6].

Vertebral artery dissecting aneurysms (VADAs) represent an uncommon variant of VAD that can be detected on angiography. Ritchey [19] reported 32 pediatric VAD, including 13 with VADAs. All 13 individuals with VADAs experienced a stroke, as confirmed by their initial imaging studies, and the rate of recurrent strokes was statistically significant within this group, affecting 9 out of the 13 patients. Among them, five patients in the VADA group underwent definitive surgical intervention following a recurrent stroke despite the initiation of medical treatment. The surgical procedures included cervical fusion for two patients, endovascular occlusion of the vertebral artery for two others, and decompression of the vertebral artery for one patient. Due to the lack of patient clinical information, these 2 cases of endovascular vertebral artery occlusion were not included in our reviewed serials. One of our patients was presented with VADA. After endovascular coils occlusion of the aneurysm and parent vessel, there was no stroke recurrence.

The efficacy of various therapeutic approaches for pediatric patients exhibiting symptoms associated with VAD remains uncertain. Hina et al. [16] employed low molecular weight heparin as primary therapy for 11 individuals, while nine patients were given aspirin. By the final follow-up, 14 individuals (comprising 70 % of the group) had a score of 0 on the modified Rankin scale (mRS), four patients (20 %) had an mRS score of 1, and three (15 %) had an mRS score of 2. Izhar et al. [2] also administered anticoagulation and antiplatelet therapy to patients in their series. Post-treatment, 16 patients achieved a full recovery. Among the remaining, 9 patients exhibited slight residual neurological impairments, and 4 patients had moderate neurological deficits. In our 2 patients and 3 of 6 cases from literatures, endovascular treatment achieved fully recovery. 3 patients from literatures remained with mild neurological deficits [6,8,9].

Endovascular treatment for VAD in the pediatric patients appears to be both secure and efficacious. Nonetheless, more extended follow-up periods and studies with larger patient cohort are necessary to confirm these preliminary findings.

## 4 Literature review

Keywords were searched in the databases of PubMed, Web of Science and Google Scholar using “pediatric vertebral artery dissection,” “pediatric vertebrobasilar dissecting aneurysm” and “endovascular treatment”. Any searchable literature concerning vertebral artery dissection and endovascular treatment in a pediatric population were included. The references cited in the articles retrieved from the search were used as secondary sources

Six cases in total reported by 6 literatures from 2001 to 2024. Available data regarding clinical, imaging characteristics, treatment and outcome are summarized in Tables 1. The disease was observed in children aged from 7 to 15 years, with an average age of 11.9 years. All of cases were male. 1 case had a history of trauma [20]. 2 cases are associated with congenital cervical anomaly [6,8]. Two cases were instances of spontaneous dissection, with no definitive causative factor identified [9,21].

**Table 1:** Summary of endovascular treated vertebral artery dissection case reports in pediatric populations.

	Year	Case	Factor	Sign	Symptom	Initial diagnosis imaging	Segment of VAD	Treatment	Outcome
Camacho [21]	2001	1	Spontaneity	Headache Vertigo	Vision change Facial nerve palsy	DSA	V3 basilar artery thrombosis	Endovascular thrombolysis	Full recovery
Sedney [6]	2011	1	Cervical abnormalities	Vomiting Vertigo Nausea	Impaired/loss of consciousness Ataxia Dysarthria	CTA DSA	V3 Dissecting aneurysm	Endovascular vertebral artery occlusion	Minor neurological deficits
Requejo [7]	2016	1				DSA	V3	Endovascular vertebral artery stent	Full recovery
Larsen [8]	2016	1	Cervical abnormalities	Headache	Vision change Hemiparesis/Hemiplegia Ataxia Dysarthria	CTA DSA	V4	Endovascular vertebral artery occlusion	Minor neurological deficits
Jia [9]	2020	1	Spontaneity	Headache		CTA MRA DSA	V4	Pipeline Embolization Devices	Full recovery
Ali [20]	2021	1	Trauma	Vomiting Nausea	Impaired/loss of consciousness Facial nerve palsy	DSA	V3 basilar artery thrombosis	Endovascular mechanical thrombectomy	Minor neurological deficits

The presence of vertebral artery dissection was evaluated by MRA in1, CTA in 3 and DSA in 6 patients. 3 cases identified posterior circulation arterial areas infarcts by MR or CT. The majority of the affected artery segments were V3, accounting for 4 out of 6 patients, with one patient involved of the V1 segment. 1 case was identified with vertebral artery dissecting aneurysms [6]. Basilar artery thrombosis was reported in 2 patients [20,21].

Headache (*n* = 3), vomiting (n = 3), nausea (n = 3), and vertigo (*n* = 2) were the most frequent clinical symptoms. Impaired/loss of consciousness (n = 3) and vision changes (n = 2) were the most common clinical abnormality detected during examination. Dysarthria (n = 2), Ataxia (n = 2), facial nerve palsy (n = 2), and Hemiparesis/hemiplegia (*n* = 1) were detected on hospital admission or developed thereafter.

The application of endovascular treatments in pediatric VAD cases, such as stent placement and angioplasty for stenotic lesions, and coiling of associated aneurysms, is quite scarce. 2 patients were managed with endovascular coils occlusion of the parent vessel. One patient was treated with endovascular vertebral artery stenting. 1 was treated with PEDs. Endovascular mechanical thrombectomy and endovascular thrombolysis was each performed in 1 patient.

Among the 6 endovascular treatment children, there were 3 fully recovery, 1 in endovascular vertebral artery stenting, 1 in endovascular thrombolysis and 1 in pipeline embolization devices. Minor neurological deficits were reported in 3 patients, including 2 in endovascular vertebral artery coils occlusion and 1 in endovascular mechanical thrombectomy.

## Author contribution

Hao Li, Bin Xu and Yanlong Tian designed the study, Yi Zhang and Yujun Liao performed the literature search and analyzed the data, Yi Zhang wrote the manuscript. All authors read and approved the manuscript.

## Clinical trial number

Not applicable.

## Consent for publication

The patient's guardian granted written consent for the publication of medical data and images.

## Ethical approval

This study was approved by The Ethics Committee of the Children's Hospital of Fudan University Children's Hospital of Fudan University ([2023] No. 332) and written informed consent was obtained from a parent of the patient.

## Guarantor

Yi Zhang

Hao Li.

## Research registration number

Not applicable.

## Funding

There is no funding support for this research.

## Conflict of interest statement

The authors declare no competing interests.

## Data Availability

All data and materials of this study are included in this published article.
